# The clinical relevance of luteal phase progesterone support in true natural cycle cryopreserved blastocyst stage embryo transfers: a retrospective cohort study

**DOI:** 10.1186/s40738-021-00096-5

**Published:** 2021-02-09

**Authors:** Ian N. Waldman, Catherine Racowsky, Emily R. Disler, Ann Thomas, Andrea Lanes, Mark D. Hornstein

**Affiliations:** Division of Reproductive Endocrinology and Infertility, Department of Obstetrics and Gynecology, Brigham and Women’s Hospital, Harvard Medical School, 75 Francis St, Boston, MA 02115 USA

**Keywords:** Luteal phase support, True natural cycle, Cryopreserved blastocyst transfer, Ongoing pregnancy, Progesterone, IVF

## Abstract

**Background:**

More than 67% of all embryos transferred in the United States involve frozen-thawed embryos. Progesterone supplementation is necessary in medicated cycles to luteinize the endometrium and prepare it for implantation, but little data is available to show if this is beneficial in true natural cycles. We evaluated the use of luteal phase progesterone supplementation for cryopreserved/warmed blastocyst transfers in true natural cycles not using an ovulatory trigger.

**Methods:**

Retrospective cohort study in a single academic medical center. We studied the use of luteal phase progesterone supplementation in patients undergoing true natural cycle cryopreserved blastocyst embryo transfers. Our primary outcome measure was ongoing pregnancy rate, with other pregnancy outcomes being evaluated (i.e. implantation rate, miscarriage rate, ectopic rate, and multifetal gestation). Categorical data were analyzed utilizing Fisher’s exact test and all binary variables were analyzed using log-binomial regression to produce a risk ratio.

**Results:**

Two hundred twenty-nine patients were included in the analysis with 149 receiving luteal phase progesterone supplementation and 80 receiving no luteal phase support. Patient demographic and cycle characteristics, and embryo quality were similar between the two groups. No difference was seen in ongoing pregnancy rate (49.0% vs. 47.5%, *p* = 0.8738), clinical pregnancy rate (50.3% vs. 47.5%, *p* = 0.7483), positive HCG rate (62.4% vs. 57.5%, *p* = 0.5965), miscarriage/abortion rate (5.4% vs. 2.5%, *p* = 0.2622), ectopic pregnancy rate (0% vs. 1.3%, *p* = 0.3493), or multifetal gestations (7.4% vs. 3.8%, *p* = 0.3166).

**Conclusion(s):**

The addition of luteal phase progesterone support in true natural cycle cryopreserved blastocyst embryo transfers did not improve pregnancy outcomes and therefore the routine use in practice cannot be recommended based on this study, but the utilization should not be discouraged without further studies.

**Capsule:**

Progesterone supplementation as luteal phase support in true natural cycle cryopreserved blastocyst transfers does not improve ongoing pregnancies.

## Introduction

As of 2017, more than two thirds of all embryo transfers in the United States involve frozen-thawed embryos with an increasing trend towards freeze-thaw cycles [1]. This trend has largely been driven by the desire to decrease ovarian hyperstimulation syndrome, the increasing use of elective single embryo transfer, the improvement in cryopreservation techniques from slow-freeze to vitrification, and the new trend towards freeze-all cycles [2].

There are currently two primary ways in which the endometrium can be prepared for embryo transfer and implantation: natural cycle and medicated prepared cycle [3]. The natural cycle takes advantage of estrogen production by the dominant follicle in the follicular phase and a subsequent shift to progesterone production by the corpus luteum after ovulation to prepare a receptive endometrium for implantation. In this setting, the cycle is referred to as a “true” natural cycle cryopreserved embryo transfer (CET) and the timing of ovulation is usually determined by Luteinizing Hormone (LH) assay. In contrast, a “modified” natural cycle CET, involves use of an exogenous ovulatory trigger, which facilitates the timing of ovulation and subsequent embryo transfer. The medicated prepared cycle involves administration of exogenous estrogen until an adequate uterine lining is achieved, and then the addition of progesterone to mimic the intrinsic switch from estrogen dominance to progesterone dominance to prepare the endometrium for implantation [3].

The decision to perform a medicated prepared cycle or a natural cycle has largely been based on physician and patient preference as there is conflicting data on the benefit of one compared with the other. Three retrospective analysis showed a benefit in performing frozen embryo transfers in natural cycles compared with medicated prepared cycles [4–6], while other retrospective analysis have shown no difference [7, 8]. Groenewoud et al. performed a prospective non-inferiority randomized controlled trial and found a non-inferior live birth rate in medicated prepared cycles compared with modified natural frozen embryo transfers. A large retrospective registry study in Sweden comparing cycles with a corpus luteum (i.e. natural cycles) compared to medicated cycles found an increase association in hypertensive disorders in pregnancy, postpartum hemorrhage, post term birth, and macrosomia were detected in medicated cycles [9]. With the most recent data showing obstetric outcome benefits to natural cycles there will likely be a shift towards this method of frozen embryo transfer.

It is therefore imperative to understand the optimal methodology to perform natural cycles. Within natural cycles the utilization of ovulatory trigger with hCG compared with spontaneous ovulation has been evaluated and also carries conflicting results. Multiple retrospective studies [10, 11] and one RCT [12] have shown no difference, while one retrospective analysis showed an improvement in clinical pregnancy rate in true natural cycles [13].

There are conflicting data reporting on the benefits of luteal phase progesterone support in true natural cycle cryopreserved embryo transfer cycles with one RCT reporting a benefit in LBR [14], while a retrospective analysis showed no difference and even a trend in clinical pregnancy rate for not supplementing in the luteal phase [13]. However, the RCT was primarily in cleavage stage embryo transfers and the retrospective analysis only evaluated clinical pregnancy rate in their primary outcome. The aim of this study was to evaluate the potential benefit for ongoing pregnancy rate with the addition of luteal phase progesterone support in true natural cycle blastocyst stage cryopreserved embryo transfers compared with no luteal phase progesterone support.

## Materials and methods

### Study population

This study is a retrospective cohort study in a single academic medical center. All patients who underwent day 5 or day 6 true natural cycle cryopreserved embryo transfers between July 30, 2012 and June 30, 2018 were screened for inclusion. Patients were excluded if they received any medication to assist in endometrial preparation, if an exogenous trigger was employed to induce ovulation, if an egg donor was utilized, if a gestation carrier was utilized, or if they received more than two embryos at time of transfer. Evaluation of outcomes following progesterone supplementation was performed and compared to those who did not receive luteal phase progesterone support. Partners Healthcare Institutional Review Board approved the study (protocol number: 2018P002957).

### Management of CET cycles

Patients initiated a true natural cycle CET in discussion with their primary provider. They called the office with cycle day 1 of their menstrual period and initiated either home urinary LH testing or serum LH testing per their prescribed protocol. If urinary LH testing, they reported a positive test and a serum progesterone was checked 3 days later to confirm ovulation with a progesterone cutoff value of 3 ng/mL. If measuring serum LH, a serum LH of 24 IU/L was taken as consistent with ovulation and no confirmatory progesterone level was collected. No further evaluation of endometrial thickness or corpus luteum was completed after this point. If luteal phase progesterone was ordered, supplementation was initiated 3 days after the LH surge. The route of progesterone administration was given based on the ordering provider’s preference and included both vaginal (Progesterone Gel 8% or Progesterone Vaginal Insert 100 mg) and intramuscular routes (Progesterone 50 mg). Embryo transfer was subsequently performed 6 days after either serum or urine LH surge with progesterone supplementation, regardless of whether embryos had been vitrified on day 5 or day 6. Progesterone was continued so long as pregnancy was viable, or until 10 gestational weeks.

### Clinical outcomes

The primary outcome was ongoing pregnancy rate defined as evidence of an intrauterine pregnancy (IUP) at discharge of care to the patient’s primary obstetrician typically around 8–12 weeks gestational age. Secondary outcomes were positive serum human chorionic gonadotropin (HCG) level, implantation rate, clinical pregnancy rate, miscarriage/abortion rate, ectopic pregnancy rate, and multifetal gestations.

Human chorionic gonadotropin (HCG) was checked 11 days after embryo transfer and trended for 3 values at 48-h intervals. A viability ultrasound was performed at 7–9 weeks gestational age or sooner if felt to be warranted by clinical indications. Implantation rate was defined as the number of gestational sacs divided by the number of embryos transferred. Clinical pregnancy was defined as one or more gestational sacs confirmed on ultrasound. Miscarriage/abortion rate was defined as the loss of pregnancy once there was confirmation of a clinical pregnancy. Ectopic pregnancy was defined as abnormally rising serum HCG quantitative values without evidence of a pregnancy on pathology after intrauterine aspiration or ultrasound confirmation of an extrauterine location. Multifetal gestation was defined as greater than one fetus ever detected on ultrasound.

Embryo quality was classified based on a modification of Gardner’s system [15], which assessed for stage based on degree of expansion (Stages 5 through 8) and quality of the inner cell mass (ICM; A through D) and trophectoderm (TE; a through d), as previously described [16]. Embryos were then classified as good, fair, or poor quality. If two embryos were transferred, the embryos were classified as the higher of the two embryo qualities.

### Statistical analyses

Values for demographic and cycle characteristics were expressed as number of patients out of all patients (percentage), and as mean ± standard deviation. For our outcomes, categorical data were analyzed utilizing Fisher’s exact test and all binary variables we used log-binomial regression to produce a risk ratio. All analyses were a priori adjusted for age. Statistical significance was assumed when the 95% confidence interval did not include 1. All statistical analyses were performed with SAS® version 9.4 software (Cary, NC, USA).

## Results

A total of 229 cycles were analyzed with 149 (65%) cycles receiving progesterone supplementation and 80 (35%) cycles receiving no progesterone supplementation. In the group receiving supplementation, no apparent differences were noted in the progesterone supplementation group compared with the control group for patient demographics as related to age, BMI, gravity, parity, infertility diagnosis, AMH, race/ethnicity, number of prior transfers at our institution, or lack of prior transfers (Table 1.)

**Table 1:** Patient Demographics

	Progesterone (*n* = 149 [65%])	No Progesterone (*n* = 80 [35%])
Age of women producing oocytes (years)	34.2 (3.9)	35.6 (3.1)
BMI at egg retrieval (kg/m^2^)	24.6 (6.1)	25.1 (6.8)
Gravity	1.4 (1.6)	1.2 (1.5)
Parous Y/N	48 (32.2%)	32 (40.0%)
Infertility Diagnosis	n (%)	
DOR	26 (17.5%)	6 (7.5%)
Unexplained	59 (39.6%)	33 (41.3%)
Uterine factor	8 (5.4%)	4 (5.0%)
Anovulatory	10 (6.7%)	3 (3.8%)
Endometriosis	9 (6.0%)	6 (7.5%)
Tubal Factor	17 (11.4%)	6 (7.5%)
Male Factor	32 (21.5%)	24 (30.0%)
AMH at start of retrieval cycle	3.4 (3.1) (*n* = 131)	4.1 (3.7) (*n* = 73)
Race/ethnicity of recipient	n (%)	
Caucasian or White	115 (77.2%)	55 (68.8%)
Black or African American	6 (4.0%)	3 (3.8%)
Hispanic or Latino/a	0	1 (1.3%)
American Indian or Alaska Native	1 (0.7%)	0
Asian	23 (15.4%)	19 (23.8%)
Other/Unknown	4 (2.7%)	2 (3.0%)
Average number of prior transfers at BWH	3.2 (3.0)	3.0 (3.4)
No prior transfers at BWH	14 (9.4%)	5 (6.3%)

Values are mean (SD)

*AMH* Antimullerian hormone, *BWH* Brigham and Women’s Hospital

Infertility diagnoses add up to more than 100% as some patients have multiple diagnoses and each was included in the analysis

Regarding cycle demographics, there was no difference in number of cryopreserved blastocyst transfers, number of fresh embryo transfers preceding the true natural cycle CET, total number of embryos transferred, number of single embryo transfers (SETs), number of donor oocyte cycles, number of ICSI transfers, total number of blastocysts frozen, or number of embryos which underwent preimplantation genetic testing for aneuploidy (PGT-A) (Table 2). There was no difference in embryo quality between the progesterone supplementation group and the control group (Table 2.).

**Table 2:** Cycle Demographics and Embryo Quality

	Progesterone (*n* = 149)	No Progesterone (*n* = 80)
Serum LH Level (IU/L)	46.1 (17.3)	51.9 (21.2)
Progesterone Level (ng/mL)	5.8 (2.7)	6.9 (2.9)
Prior CET cycles	1.3 (0.6)	1.3 (0.7)
Fresh embryo transfers preceding true natural cycle CET	135 (90.6%)	75 (93.8%)
Average number of blastocysts transferred	1.3 (0.5)	1.2 (0.4)
Total number of SETs	105 (70.5%)	68 (85.0%)
Number of donor oocyte cycles	5 (3.4%)	0 (0.0%)
Number of cycles using ICSI	62 (41.6%)	39 (48.8%)
Average number of blastocysts frozen	5.1 (3.5)	5.6 (3.9)
Number of cycles using PGT-A	25 (16.8%)	7 (8.8%)
Number of cycles with > 1 good-quality blastocyst^a^ transferred	47 (31.6%)	30 (37.5%)
Number of cycles with no good quality and > 1fair-quality embryo^a^	48 (32.2%)	28 (35.0%)
Number of cycles with only poor-quality embryo(s) transferred	54 (36.2%)	22 (27.5%)

Values are mean (SD) or number (%)

^a^If multiple embryos transferred it was reported as the better quality embryo if not the same

When comparing cycles supplemented with progesterone to those without supplementation, no difference was observed in the percentage of cycles with a positive HCG, clinical pregnancy, miscarriage/abortion, ectopic pregnancy, multifetal gestation, or ongoing pregnancy (Table 3).

**Table 3:** Clinical Outcomes

	Progesterone Supplementation (*n* = 149)	No Progesterone Supplementation (*n* = 80)	Unadjusted Statistics (*p* value and risk ratio*)	Age-Adjusted Statistics
Positive hCG	93 (62.4%)	46 (57.5%)	0.5039 1.08 (0.86, 1.37)	0.5965 1.07 (0.84, 1.35)
Clinical pregnancy	75 (50.3%)	38 (47.5%)	0.6592 1.07 (0.80, 1.42)	0.7483 1.05 (0.79,1.39)
Miscarriage / Abortion	8 (5.4%)	2 (2.5%)	0.2918 2.21 (0.51, 9.68)	0.2622 2.33 (0.53, 10.24)
Ectopic pregnancy	0 (0.0%)	1 (1.3%)	0.3493	(no further modeling)
Multifetal gestation	11 (7.4%)	3 (3.8%)	0.2835 1.98 (0.57, 6.95)	0.3166 1.87 (0.55, 6.36)
Ongoing Pregnancy	73 (49.0%)	38 (47.5%)	0.7871 1.04 (0.78, 1.39)	0.8738 1.01 (0.76, 1.34)

Values are total number (%) or *p*-value and age-adjusted risk ratio with 95% CIs

## Discussion

The major finding of this study was that no statistical difference was observed in the incidence of ongoing pregnancies with luteal phase progesterone supplementation in true natural cycle cryopreserved blastocyst transfers compared with transfer not supplemented with progesterone. Also, no difference between the two groups was found regarding the incidence of cycles resulting in positive HCG, implantation rate, clinical pregnancy, miscarriage/spontaneous abortion, ectopic pregnancy, or multifetal gestation.

Our finding of no difference between the two groups for ongoing pregnancy contrast that of a significantly higher live birth rate reported by Bjuresten et al. in true natural cycle CET cycles supplemented with vaginal progesterone in a randomized controlled trial (30% vs. 20%, *P* = 0.0272). Similar to our findings for the other clinical outcomes, however, Bjuresten et al. did not find a difference in the percentage of cycles with a positive HCG, clinical pregnancy, miscarriage, or spontaneous abortion [14]. The primary difference between our study and the Bjuresten et al. study is that we included only blastocyst transfers whereas Bjuresten et al. included nearly entirely cleavage stage transfers, perhaps making whatever benefit was seen only persist for cleavage stage transfers. Another difference between our two study populations was that we included all types of progesterone supplementation and not just vaginal, which could be a possible reason for variation in our study findings.

A similar large retrospective study evaluated vaginal progesterone luteal phase support for 2 weeks after day 3 and day 5 transfers and found a decrease in live birth rate for those true natural CET cycles without progesterone luteal phase support, compared with those receiving progesterone luteal phase support with an odds ratio of 0.58 (*p* = 0.003) [17].

Lee et al. performed a RCT looking at true natural cycle CET of cleavage stage embryos with HCG luteal phase support and found no difference in regard to ongoing pregnancy rate, implantation rate, or miscarriage rate [18]. This is consistent with our findings that luteal phase support in true natural cycle CET does not offer clinical improvement. We did not evaluate HCG as a means of luteal phase support, but no correlation in either study was seen perhaps indicating a lack of benefit regardless of type of luteal phase support utilized.

Several subsequent studies evaluated luteal phase support in modified natural cycle CETs with an HCG trigger. Eftekhar et al. performed a RCT of luteal phase support with IM progesterone on modified natural cycle with HCG trigger and found no difference in clinical pregnancy rate, implantation rate, or spontaneous abortion rate [19]. Two other retrospective studies evaluated modified natural cycles with vaginal progesterone and found no difference in ongoing pregnancy rate [20], clinical pregnancy rate, implantation rate, and multiple pregnancy rate, but Kim et al. did find a decrease in miscarriage rate and improvement in LBR with progesterone supplementation [21]. Lee et al. performed a retrospective study which did not find an improvement in luteal phase support with HCG injections on implantation rate, clinical pregnancy rate, or miscarriage rate [22]. A recent meta-analysis evaluating natural cycles with vaginal progesterone for luteal phase support was completed which included 1 RCT (Bjuresten et al.) and 3 retrospective studies also confirmed there is no association between vaginal progesterone for luteal phase support and CPR [23].

As can be seen by the variation in findings of our study and those that preceded ours, the debate as to whether progesterone luteal phase support is beneficial in true natural cycles persists and no large practice changes should be made based on the findings of our study as it is a retrospective analysis and only associations can be inferred. There are clear benefits and deficits to both medicated prepared cycles and natural cycles. The primary benefit to the medicated prepared cycle is the ability to control the timing of the cycle but requires patients to take daily medication and often intramuscular progesterone injections until the luteal placental shift occurs. A seemingly more patient friendly endometrial preparation is the natural cycle where patients take advantage of the endogenous estrogen production and progesterone production by the corpus luteum after ovulation. A large issue with this type of cycle is the need for regular LH monitoring and lack of control over the timing of the cycle and need for transfer. As more data comes out on the potential benefits of having a corpus luteum, so too must we continue to optimize the cycle preparation and potential need for LPS.

Collectively, our study adds to the literature reporting no difference in ongoing pregnancy with luteal phase support in true natural cycle blastocyst cryopreserved transfers. However, there is discrepancy in the literature in which patients might benefit from luteal phase support which needs further study. It would seem the presence of a corpus luteum and the absence of a true luteal phase defect would indicate the lack of need for progesterone supplementation, particularly in blastocyst stage transfers. At this time, we can neither support nor strongly refute giving luteal phase support.

The primary strength of this paper is that this is the first study to evaluate luteal phase progesterone supplementation in true natural cycle blastocyst cryopreserved transfers. The limitations of the study are its retrospective design, modest study size, no single regimen by which the luteal phase support was given although we did control for the regimen used, and the primary outcome was ongoing pregnancy not live birth rate. Future studies addressing these issues would certainly be warranted.

## Conclusion

In true natural cycle blastocyst cryopreserved transfers there does not appear to be a benefit to giving progesterone luteal phase support regarding ongoing pregnancy, or the incidence of cycles resulting in a positive HCG, clinical pregnancy or an increase in the implantation rate.

## Data Availability

The datasets used and/or analysed during the current study are available from the corresponding author on reasonable request.
